# The more the better? The non-linear effect of workload on workplace well-being

**DOI:** 10.3389/fpsyg.2026.1746071

**Published:** 2026-01-21

**Authors:** Juan Liang, Wenxin Zhou, Bibo Xu, Hai Jiang

**Affiliations:** 1 Department of Psychology, Hubei University, Wuhan, Hubei, China; 2 Party School of CPC Wuhan Committee, Wuhan, Hubei, China

**Keywords:** autonomous motivation, experience sampling methodology (ESM), non-linear effects, workload, workplace well-being

## Abstract

**Introduction:**

It is unclear what level of workload is best for workplace well-being, in part because researchers have only tested linear relationships. We tested an inverted U-shaped model in which workplace well-being is optimal at a moderate level of workload. The mediation effect of autonomous motivation were also tested.

**Methods:**

We conducted two studies with employees. Study 1 (*N* = 324) used a cross-sectional survey design and was analyzed using SPSS20.0. Study 2 (*N* = 152) used experience sampling methodology and was analyzed using Mplus8.3. The data were analyzed using non-linear analytical techniques and mediation analyses conducted with the PROCESS macro.

**Results:**

The data were analyzed using non-linear analytical techniques and mediation analyses conducted with the PROCESS macro. The results of both studies showed a U-shaped curve, but contrary to expectations, the curve was positive rather than inverted: an increase in workload from low to moderate was associated with decreased workplace well-being, but an increase in workload from moderate to high was associated with increased workplace well-being. The mediation effect of autonomous motivation emerged in study 1, but not Study 2.

**Discussion:**

These results suggest that employees should be given a moderate workload that can be increased, rather than starting with a low workload.

## Introduction

A person’s strengths in the work environment, such as productivity, adaptability, and endurance, are called workplace well-being (Diener and Chan, 2011; Huang and Peng, 2015). Higher workplace well-being has been shown to be related to higher psychological adjustment (Diener and Chan, 2011) and better work performance (Baas et al., 2008). Many studies have examined whether workload affects employees’ workplace well-being, but results have been mixed. Workload has been shown to have a detrimental effect on employees’ well-being at work in some studies (Cropley et al., 2020; Fritz and Sonnentag, 2006; Ilies et al., 2010; Kuijpers et al., 2020; Geurts et al., 2003), but to have a positive effect in other research (Lindfors et al., 2006; Lundberg and Frankenhaeuser, 1999).

There are two gaps in the literature on workload and workplace well-being. Studies to date have assumed and only tested a linear relation between workload and workplace well-being. In the current research, we challenge the assumption of linearity and propose that the effect of workload is in fact non-linear: At the low end of the spectrum, increasing the workload should promote workplace well-being; at the high end of the spectrum, increasing workload could have a detrimental effect on workplace well-being. In addition, previous research has been inconclusive about the mechanism by which workload affects workplace well-being. We address these gaps by testing a model in which autonomous motivation mediates a non-linear relationship between workload and workplace well-being.

### Relationship between workload and workplace well-being

Research on the relationship between workload and workplace well-being has yielded inconsistent findings. Some studies have suggested that a high workload might negatively impact an employee’s workplace well-being. Workload could increase employees’ emotional distress (Ilies et al., 2010), burnout (Kuijpers et al., 2020), and exhaustion (Cropley et al., 2020), which in turn could undermine their workplace well-being. For example, using an experience-sampling methodology, Ilies et al. (2010) found that workload was positively associated with affective distress, blood pressure, as well as other indicators of lower daily well-being. Geurts et al. (2003) conducted two cross-sectional studies to examine the relationships among workload, work–home interference, and two indicators of employee well-being (i.e., work-related negative affect and health complaints). They found that work–home interference mediated the association between workload and employee well-being. Based on these results, the authors argued that workload undermines well-being through a spillover process in which work-related strain extends into non-working hours, limiting opportunities for adequate recovery (Geurts et al., 2003).

However, a high workload may also promote workplace well-being (Lindfors et al., 2006). A high workload helps an employee’s sense of purpose, positive interpersonal relationships, positive self-perception, and sense of autonomy (Lindfors et al., 2006; Lundberg and Frankenhaeuser, 1999). Using a cross-sectional design, Lindfors et al. (2006) showed that longer hours of paid work were associated with higher levels of personal growth, one of the dimensions of well-being examined in their study. High workload has been shown to be uncorrelated with negative emotions such as depression (Spector and Jex, 1998). Pace et al. (2021) examined the relationship between university professors’ workload and workplace well-being, showing that workload tied to bureaucratic university practices negatively affects their perception of work-related well-being.

Is there a purely linear gain or loss relationship between workload and workplace well-being? Based on self-determination theory, we suggest that there may be a curvilinear relationship between workload and workplace well-being. That is to say, the relationship between the two may show an inverted U-shaped curve.

### Workload and workplace well-being: proposal for a non-linear relation

The self-determination theory (SDT; Deci and Ryan, 2000) provides the conceptual framework for our proposal that workload is non-linearly related to workplace well-being. SDT posits that human beings have three fundamental psychological needs: autonomy, competence and relatedness. According to this theory, satisfaction of fundamental psychological needs indispensable for an individual’s psychological growth, integrity, and well-being (Bradshaw et al., 2025; Clements and Rostosky, 2025; Hindman et al., 2025; Kuykendall et al., 2020). When individuals are in environments that fulfill of their fundamental psychological needs, they are more likely to flourish and experience a heightened sense of well-being (Deci and Ryan, 2000). Conversely, environments that fail to fulfill fundamental psychological needs impede individual development and undermine well-being (Deci and Ryan, 2000).

The workplace is an environment that could provide opportunities to learn and grow (Cavanaugh et al., 2000; Slemp et al., 2020) potentially meets the fundamental psychological need, such as, competence (Deci and Ryan, 2000). Through work, employees can develop self-worth and engage in personal growth (Li et al., 2015), and the meaningfulness of work may further foster employees’ autonomy (Gagné and Deci, 2005). In addition, social support in the workplace satisfies the need for relatedness (Bakker and Demerouti, 2007; De Cooman et al., 2013). We propose that this potential is most likely to be realized when employees have a moderate workload. At moderate levels of workload, employees are more likely to complete challenging tasks, which enables them to experience a sense of achievement (Kc et al., 2020) and competence. Under such conditions, they also have the time and energy to interact with colleagues, enabling the mutual exchange of social support. This reciprocal process fulfills their need for relatedness (Bakker and Demerouti, 2007; De Cooman et al., 2013; Zeijen et al., 2020). This proposal is consistent with previous research. Kc et al. (2020) showed that individuals assigned a moderate workload were productive and gained a sense of achievement from task completion. Feng et al. (2017) found that moderate to high levels of working time pressure enhanced the employees’ well-being. In a similar vein, we expect that a moderate workload will be associated with maximum workplace well-being.

By contrast, workloads that are too low or too high should undermine workplace well-being. Employees with low workloads tend to view their work as unchallenging and boring (Deci and Ryan, 2000), which should interfere with their needs for autonomy and competence being met (Gagné et al., 2000; Karasek, 1979). Low workloads restrict employees’ opportunities to interact with colleagues. Such limited interpersonal engagement precludes the satisfaction of their need for relatedness and deprives them of the social feedback necessary to assess the person’s current level of affiliation (O’Connor and Rosenblood, 1996; Weick, 1979; Weiss, 1973). Consequently, the absence of these signals heightens the degree to which employees perceive their basic psychological need for relatedness as unfulfilled (Tang et al., 2023). At the other extreme, employees may be unable to cope with a high workload. A high workload may require work during non-working time, which would frustrate their need for autonomy (Dettmers et al., 2016). In addition, a high workload may lead to spend less time for family and friends (Ward et al., 2020) which may undermine employees’ relatedness needs. Furthermore, an excessive workload may engender a sense of failure, which in turn may diminish self-efficacy (Evans, 1989), which may undermine employees’ competence. Hence, the lower or higher workloads often interferes with fulfilling basic psychological needs for autonomy, competence, and relatedness, impairing employees’ workplace well-being.

*H1*: Workload has an inverted U-shaped relationship with workplace well-being. That is, at a moderate level of workload, an individual's workplace well-being is higher than at a low or high level of workload.

### The mediating role of autonomous motivation

We propose that autonomous motivation (i.e., being motivated by the inherent value of an activity or the inherent enjoyment of an activity; Deci and Ryan, 2000) mediates the association between workload and workplace well-being. According to SDT, satisfaction of fundamental psychological needs would invoke autonomous motivation, which is more adaptive for well-being (Deci and Ryan, 2000). The association between workload and autonomous motivation, can be conceptualized in two ways. First, an optimal workload creates a “flow” experience for individuals (Csikszentmihalyi, 1975), which is a prototype for autonomous motivation (Deci and Ryan, 2000). Second, employees may view a moderate workload as promoting their personal growth and achievement, which may help employees find meaning in the work and then promote autonomous motivation (Gagné et al., 2000). In addition, the demands of a moderate workload do not interfere with employees’ needs outside of work, and the employee’s needs for autonomy and relatedness are met (Lindfors et al., 2006). On the one hand, a moderate level of workload can satisfy individuals’ needs for relatedness, competence, and autonomy, thereby further stimulating their autonomous motivation. On the other hand, the moderate workload itself can directly activate autonomous motivation.

By contrast, a limited workload (below the optimal level) would be associated with lower autonomous motivation because it impedes employees’ understanding the meaningfulness in the work. Understanding the rationale and meaningfulness behind the task is one of important factors supporting autonomy (Gagné et al., 2000). When the workload is below an optimal level, employees may have fewer opportunities to acquire work information, knowledge, and skills, and so experience fewer opportunities to develop their competence. This should engender lower meaningfulness and self-efficiency, which in turn would lead to lower autonomous motivation (Gagné et al., 2000). As noted earlier, a low workload limits individuals’ interactions with colleagues, reducing opportunities for the mutual exchange of social support and thereby hindering the fulfillment of their need for relatedness. Consequently, a low workload undermines the satisfaction of basic psychological needs, which in turn diminishes autonomous motivation. Similarly, a high workload (above the optimal level) may impose excessive work demands and be out of the person’s control (Pace et al., 2021), which may interfere with non-work life and are likely to limit employees to recover (Kuykendall et al., 2020). That may also undermine autonomy and relatedness (Gagné et al., 2000). When individuals devote most of their time to work and experience pressure to meet performance goals, they have limited opportunities to cultivate high-quality interpersonal relationships with friends and colleagues (Ward et al., 2020), thereby hindering the fulfillment of their need for relatedness. Thus, both excessively low and high workloads hinder the fulfillment of individuals’ basic needs, which undermines autonomous motivation. This diminished autonomy, in turn, compromises their well-being.

*H2*: Autonomous motivation mediates the inverted U-shaped curve relationship between workload and workplace well-being.

## Method

We conducted two studies to test a non-linear relationship between workload and workplace well-being, the mediating function of autonomous motivation. The research was approved by the research ethics committee of the university with which the first author is affiliated. The volunteer participants in both studies were adults employed in China. In Study 1, participants completed questionnaires in a cross-sectional design (*N =* 324). In Study 2, experience sampling data were collected (760 observations from *N* = 152 participants). All research materials, data, and analysis scripts are available on the Open Science framework (OSF): https://osf.io/672xc/?view_only=15b146b2074240afbf2de48d4408bf4c.

### Study 1

#### Participants

We used convenience sampling. University students helped recruit participants by sharing an online questionnaire with their employed relatives and friends. All participants were required to be currently working. The participants were employees in a range of sectors including healthcare, education, and industry located in Beijing, Guangdong, Hubei, Hunan, Inner Mongolia, Jiangsu, and other cities in China. Three hundred twenty-seven volunteered to participate and completed the questionnaires. Because three participants gave the same rating on 1 or 5, the final sample consisted of 324 individuals (*M*_age_ = 36.15, *SD*_age_ = 9.82; *M*_job tenure_ = 2.75, *SD*_job tenure_ = 1.17; 56.2% men).

#### Measures

##### Workload

We measured workload with the three-item Work Experience and Evaluation Questionnaire adapted from the Job Content Questionnaire (Hülsheger et al., 2018). Participants rated the items on this scale from 1 = *do not agree at all* to 4 = *fully agree* (sample item: “I have too much work to do”). Cronbach’s alpha was 0.75.

##### Workplace well-being

We measured workplace well-being with the six-item Workplace Well-Being subscale of the Employee Well-Being scale (Zheng et al., 2015). Participants rated the items from 1 = *do not agree at all* to 5 = *fully agree* (sample item: “I am satisfied with my work responsibilities”). Cronbach’s alpha was 0.91.

##### Autonomous motivation

We measured autonomous motivation with the six-item autonomy motivation subscale of the Work Motivation Scale (Gagné et al., 2010). Participants rated the items from 1 = *none or almost none of the time* to 5 = *all or almost all of the time* (sample item: “Because this job fits my personal values”). Cronbach’s alpha was 0.76.

##### Control variables

We used the following socio-demographic and economic variables as covariates: gender, age, job tenure, monthly salary level. Prior research on the relationship between workload and workplace well-being has often adopted the conservation of resources theory. To control for relevant variables from this theoretical perspective, emotional exhaustion was included as a control variable (Li and Shi, 2003). We used the Emotional Exhaustion subscale from the Maslach Burnout Inventory (MBI; Maslach and Jackson, 1981). Participants rated the items from 1 = *almost none of the time* to 7 = *all or almost all of the day* (sample item: “I’m physically and mentally exhausted from work”). Cronbach’s alpha was 0.91.

#### Analytic plan

We used hierarchical regression to test non-linear effect of workload on workplace well-being. If the squared term for workload was significant, it would indicate a non-linear effect.

We adopted the approach of Hayes and Preacher (2010) to test the mediation effect of non-linear model. Specially, we estimated the instantaneous indirect effect of autonomous motivation at “relatively low,” “relatively moderate,” and “relatively high” level workload.

Common method bias was tested using Harman’s single-factor test. There were multiple common factors with eigenvalues greater than 1, and the first factor explained 39.75% of the total variance (<40%). It can thus be concluded that there was no serious common method bias problem in this study.

#### Results

##### Descriptive statistics

Table 1 shows the means, standard deviation and correlation of all variables. Correlational analyses indicated that workload was positively correlated with emotional exhaustion (*r =* 0.38, *p < 0*.001). Autonomous motivation was positively correlated with workplace well-being (*r =* 0.75, *p <* 0.001) and negatively correlated with emotional exhaustion (*r =* −0.35, *p <* 0.001). Furthermore, workplace well-being was negatively correlated with emotional exhaustion (*r =* −0.38, *p <* 0.001).

**Table 1:** Results of hierarchical multiple regression models testing the inverted U hypotheses in Study 1.

Variable	Model 1			Model 2			Model 3			Model 4		
	*b*	SE	95%CI	*b*	SE	95%CI	*b*	SE	95%CI	*b*	SE	95%CI
Gender	0.03	0.08	(−0.13, 0.18)	0.03	0.08	(−0.13, 0.19)	0.06	0.08	(−0.10, 0.22)	−0.001	0.06	(−0.11, 0.11)
Age	0.02^***^	0.01	(0.01, 0.03)	0.02^***^	0.01	(0.01, 0.08)	0.02^***^	0.01	(0.01, 0.03)	0.01^**^	0.00	(0.00, 0.02)
Job tenure	−0.02	0.04	(−0.10, 0.07)	−0.02	0.04	(−0.10, 04)	−0.02	0.04	(−0.09, 0.06)	−0.02	0.03	(−0.08, 0.04)
Monthly salary level	0.01	0.02	(−0.03, 0.04)	0.01	0.02	(−0.03, 0.04)	0.01	0.02	(−0.03, 0.04)	0.01	0.01	(−0.01, 0.04)
Emotional exhaustion	−0.25^***^	0.04	(−0.32, −18)	−0.26^***^	0.04	(−0.34, −0.18)	−0.27^***^	0.04	(−0.35, −19)	−0.10^**^	0.03	(−0.16, −0.04)
Workload				0.05	0.08	(−0.10, −0.20)	0.05	0.08	(−0.10, 0.20)	−0.02	0.05	(−0.12, 0.10)
Quadratic term of workload							0.27^**^	0.08	(0.12, 0.43)	0.14^*^	0.06	(0.03, 0.25)
Autonomous motivation										0.63^***^	0.04	(0.56, 0.71)

*N* = 324. Unstandardized regression coefficients are shown. + *p* < 0.1, ** *p* < 0.01, *** *p* < 0.001.

##### Hypothesis 1: the inverted U-shaped curve relationship between workload and workplace well-being

**Figure 1:**
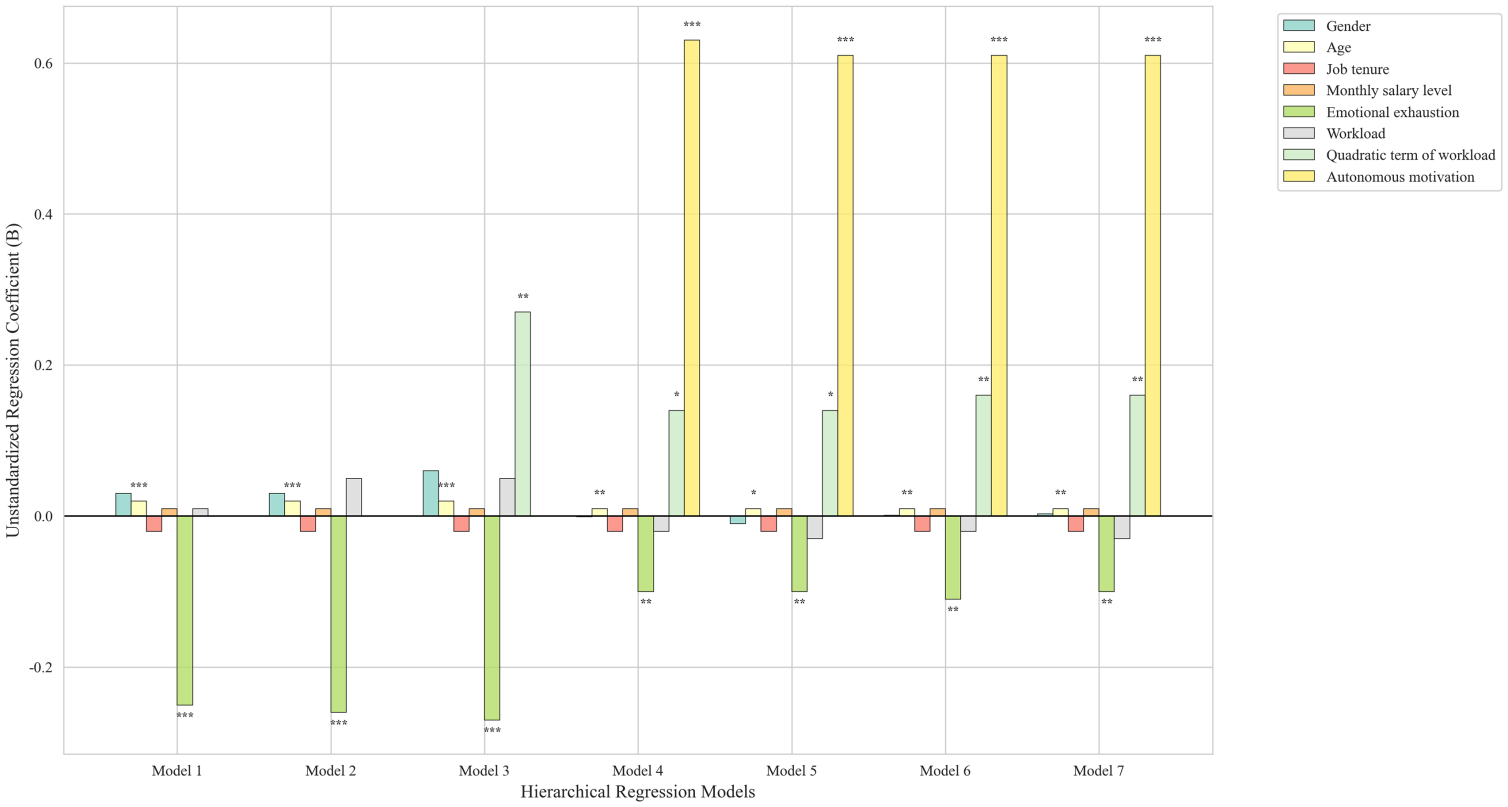
Results of hierarchical multiple regression testing the inverted U hypotheses in Study 1.

**Figure 2:**
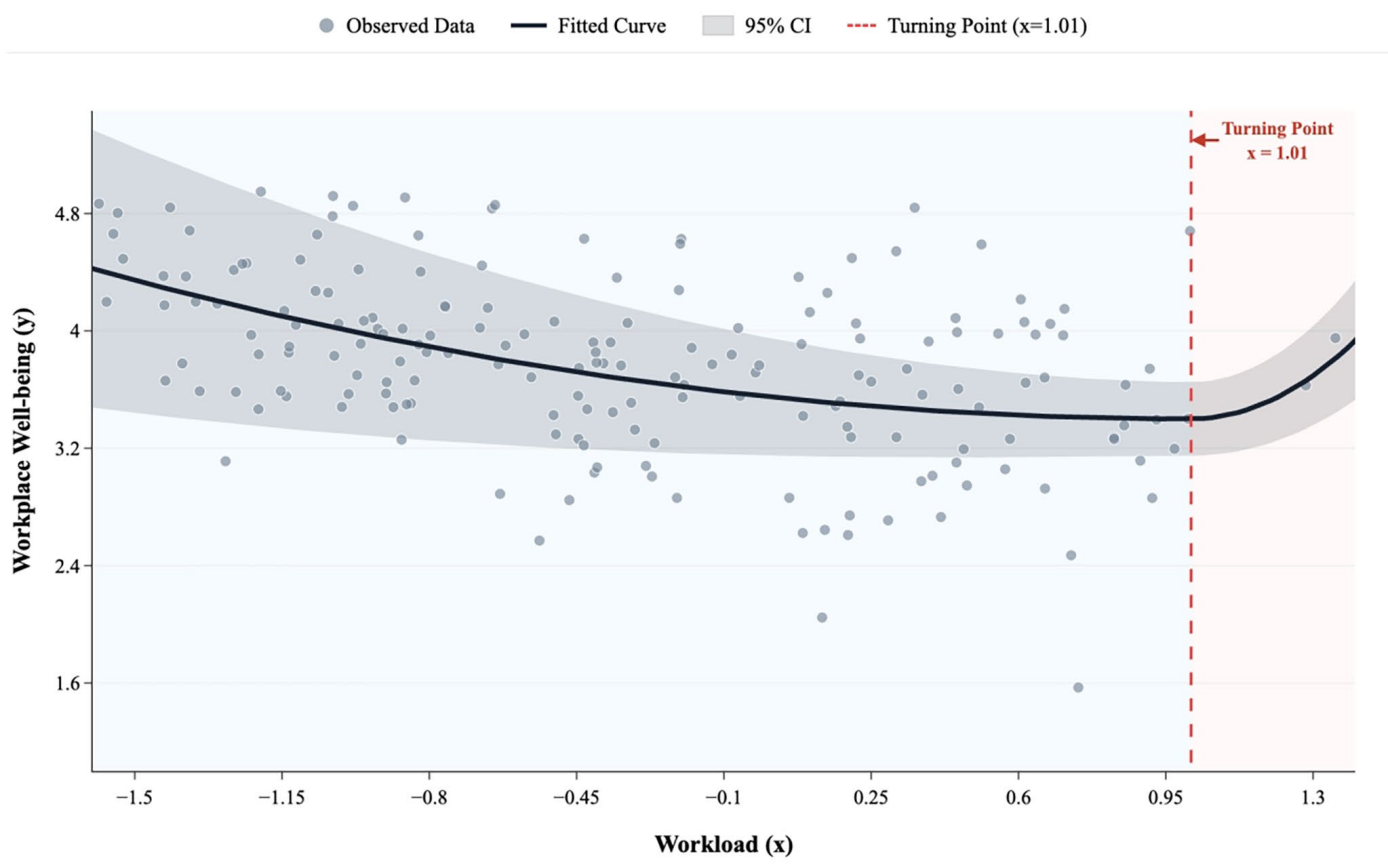
Positive U-curve relationship between workload and workplace well-being found in Study 1.

To examine the non-linear relationship between workload and workplace well-being, we estimated a quadratic regression model including both the linear and squared terms of workload (see Table 2 and Figure 1). The linear term for workload on workplace well-being was not significant (*b* = 0.05, *p* > 0.05). However, the quadratic term for workload on workplace well-being was significant (*b =* 0.27, *p* < 0.01), providing support for the non-linear effect of workload on workplace well-being. Contrary to Hypothesis 1, the relationship between workload and workplace well-being showed a positive U-shaped relationship rather than an inverted U-shaped one, such that increasing workload from low to moderate decreased workplace well-being, but increasing workload from moderate to high improved workplace well-being (see Figure 2).

**Table 2:** Means, standard deviations, and correlations among variables in Study 1.

	*M*	*SD*	1	2	3	4	5	6	7
1 Age	36.15	9.82	1						
2 Length of service	2.75	1.17	0.50^***^	1					
3 Monthly salary level	6.13	2.54	0.11	0.35^**^	1				
4 Workload	2.66	0.56	0.06	0.03	−0.02	1			
5 Autonomous motivation	3.63	0.82	0.25^***^	0.12^*^	−0.02	−0.05	1		
6 Workplace well-being	3.63	0.77	0.28^***^	0.12^*^	0.03	−0.09	0.75^***^	1	
7 Emotional exhaustion	3.00	1.05	−0.18^**^	−0.06	0.03	0.38^***^	−0.35^***^	−0.38^***^	1

*N* = 324. ^*^p < 0.05, ^**^p < 0.01, ^***^p < 0.001.

##### Hypothesis 2: the mediation effect of autonomous motivation

Bootstrapped analyses of the conditional indirect effects indicated that the mediation effect of autonomous motivation varied across levels of workload. Specifically, at a high level of workload, that is one standard deviation above the mean (*X* = 0.56), the indirect effect was positive and significant (*θ*_*x* = 0.56_ = 0.23, 95% CI = [0.03, 0.37]). At a moderate level of workload, that is the mean (*X* = 0), the indirect effect was not statistically significant (*θ*_*x* = 0_ = 0.07, 95% CI = [−0.06, 0.19]). At a low level of workload, that is one standard deviation above the mean (*X* = −0.56), the indirect effect was non-significant (*θ*_*x* = −0.56_ = −0.09, 95% CI [−0.31, 0.10]). Hence, increasing workload among employees who are relatively low in workload would slightly decrease workplace well-being via its effect on autonomous motivation, but increasing workload among employees who are already moderate to high would increase workplace well-being through autonomous motivation.

Importantly, the mediation effect showed an incremental increase from the low to medium to high workload conditions (−0.09 < 0.07 < 0.23), suggesting that instantaneous indirect effect is non-linear in workload (Ye et al., 2023). Although, the relationship between workload and autonomous motivation was a positive U-shape, these results did not support Hypothesis 2.

#### Supplementary analyses 1: spline and piecewise checks

To further validate the non-linear relationship between workload and workplace well-being, we conducted restricted cubic spline and piecewise regression analyses using R (version 4.0.0).

##### Restricted cubic spline analyses

We first estimated a linear baseline model including workload and control variables. We then estimated models incorporating restricted cubic spline terms for workload. Likelihood ratio tests indicated that the spline model with four-degree-of-freedom fit the data significantly better than the linear model, *F*(3, 314) = 5.27, *p* = 0.001, providing strong evidence for a non-linear association between workload and workplace well-being.

Model fit comparisons further showed that the four-degree-of-freedom spline model exhibited the best fit (*R*^2^ = 0.23, adjusted *R*^2^ = 0.21, AIC = 682.45, BIC = 724.04), outperforming the linear specification (*R*^2^ = 0.20, adjusted R^2^ = 0.18, AIC = 692.37, BIC = 722.62) as well as alternative spline specifications. In addition, generalized additive model (GAM) analyses yielded convergent results, with a significant smooth term for workload (edf = 2.37, *F* = 3.37, *p* = 0.021). Together, these findings consistently support the presence of a non-linear relationship between workload and workplace well-being.

Piecewise Regression and Turning Point Identification. To further examine the shape of this non-linear relationship, we conducted piecewise regression analyses to identify potential turning points. For the relationship between workload and workplace well-being, a statistically significant turning point was identified at workload = 1.01 (95% CI [0.88, 1.01]). The piecewise model explained significantly more variance than the linear model, *F*(2, 315) = 9.44, *p* < 0.001 (*R*^2^ = 0.24).

Before the turning point, the effect of workload on workplace well-being was negative but not statistically significant (*β* = −0.13, *SE* = 0.09, 95% CI [−0.31, 0.06]). After the turning point, workload exerted a strong and significant positive effect on workplace well-being (*β* = 2.99, *SE* = 0.82, 95%CI [1.38, 4.59]). This pattern indicates a reversal in the direction of the relationship, consistent with a positive U-shaped association.

##### Piecewise mediation analyses

Parallel piecewise analyses were conducted for the relationship between workload and autonomous motivation. A similar turning point was identified at workload = 1.01. Prior to this threshold, workload was weakly and non-significantly related to autonomous motivation (*β* = −0.03, *SE* = 0.09, 95% CI [−0.20, 0.14]). Beyond the turning point, workload showed a strong and significant positive association with autonomous motivation (*β* = 2.45, *SE* = 0.35, 95% CI [1.76, 3.14]).

Across the entire sample, autonomous motivation was a stable and strong predictor of workplace well-being (*b* = 0.64, *p* < 0.001).

Conditional indirect effects further revealed a pronounced threshold pattern. The indirect effect of workload on workplace well-being via autonomous motivation was non-significant below the turning point (indirect effect = −0.02, 95% CI [−0.13, 0.09]) but became large and statistically significant above the turning point (indirect effect = 1.58, 95% CI [1.13, 2.03]). After accounting for autonomous motivation, the direct effect of workload on workplace well-being was no longer significant, and the total effect remained non-significant. These results suggest that, beyond the identified threshold, the positive association between workload and workplace well-being operates primarily through autonomous motivation.

#### Supplementary analyses 2: robustness

To examine the robustness of the non-linear relationship between workload and workplace well-being, we conducted additional analyses without controlling for emotional exhaustion. Results from the hierarchical regression model showed that, in the model excluding emotional exhaustion, the quadratic term of workload remained significant and positive (*b =* 0.24, *p =* 0.004), consistent in both direction and significance with the primary model that included emotional exhaustion. This indicates that the U-shaped relationship between workload and workplace well-being is robust and not an artifact of over-controlling for emotional exhaustion.

In mediation tests, the model with autonomous motivation as the dependent variable also showed a significant quadratic effect of workload (*b =* 0.18, *p =* 0.045). We further tested the indirect effects using the medcurve macro. The conditional indirect effects at relatively low and high workload levels (*θ_X = −0.56_* = −0.20, 95% CI [−0.20, −0.06]; *θ_X = 0.56_* = 0.07, 95% CI [0.07, 0.24]) were consistent with those obtained in the primary models.

Study 1 used a cross-sectional sample to provide an initial examination of the relationships among workload, autonomous motivation, and workplace well-being. However, individuals’ workload may fluctuate within persons over time; that is, workload can vary across workdays. How, then, do day-to-day variations in workload affect autonomous motivation and workplace well-being? To address this question, Study 2 employed an experience sampling methodology to further examine and validate the relationships among workload, autonomous motivation, and workplace well-being.

### Study 2

In Study 2, we investigated the non-linear effect of workload on workplace well-being by using experience sampling methodology over the course of five consecutive workdays. A convenience sampling method was used to recruit employed participants, and efforts were made to avoid overlap with Study 1. We required participants to start on Monday and end on Friday. On the first day, participants reported demographic information, workload, autonomous motivation, emotional exhaustion, and workplace well-being. On each of the four subsequent days, they reported daily workload, autonomous motivation, emotional exhaustion, and workplace well-being.

#### Participants

We recruited employees in China online using snowball sampling in various social networks. Overall, 219 participants (31 recruited directly by the author team and 188 via snowball) who completed the first day’s survey. And 152 who completed at least 4 days of daily surveys (*M*_age_ = 29.03, *SD*_age_ = 8.75; *M*_job tenure_ = 2.29, *SD*_job tenure_ = 1.03; 47.4% men). We handled missing data in Day 5 using a person-mean score for this participant across the 4-day period. Finally, our analyses were based on 760 observations. Participants received 8 CNY for their at least 4 days of daily surveys completions.

#### Procedure and measures

Participants received a link to a daily assessment every day for 5 days. The link was sent at 6 p.m. Beijing Standard Time and was active for 6 h. We send our link to 31 participants via WeChat. We did not directly contact the rest of participants; instead, we ask these 31 participants to send our link to the rest of participants they inviting.

We measured the same variables as in Study 1, but added wording to emphasize what day it was. Over the 5 days, the Cronbach’s *α* for workload were 0.81 to −0.86, autonomous motivation were 0.89 to −0.93, workplace well-being were 0.89 to −0.92, and emotional exhaustion were 0.90 to −0.96.

#### Analytic plan

We used Mplus 8.3 to text our hypotheses. The null model results for workplace well-being showed ICC = 0.63, indicating that our sample was suitable for testing a multilevel model. Before analysis, we centered workload and autonomous motivation on the group means.

#### Results

##### Descriptive statistics

Table 3 shows the means, standard deviations, and correlations among all variables. Correlational analyses revealed that workload correlated positively with emotional exhaustion (*r =* 0.56, *p <* 0.001) and negatively with workplace well-being (*r =* −0.10, *p = 0*.005). Autonomous motivation correlated positively with workplace well-being (*r =* 0.72, *p <* 0.001) and negatively with emotional exhaustion (*r =* −0.22, *p <* 0.001). Workplace well-being also correlated negatively with emotional exhaustion (*r =* −0.31, *p <* 0.001).

**Table 3:** Means, standard deviations, and correlations among variables in Study 2.

	*M*	*SD*	1	2	3	4	5	6	7	8	9	10	11	12	13	14	15	16	17	18	19	20	21	22	23
1 Age	29.03	8.75	1																						
2 Length of service	2.29	1.03	0.68^***^	1																					
3 Monthly salary level	6.76	2.47	−0.01	0.21^**^	1																				
4 Day 1 workload	2.55	0.65	0.22^**^	0.20^*^	−0.18^*^	1																			
5 Day 1 autonomous motivation	3.16	0.83	0.16^*^	0.14	0.03	−0.01	1																		
6 Day 1 workplace well-being	3.36	0.84	0.09	0.10	0.06	−0.12	0.76^***^	1																	
7 Day 1 emotional exhaustion	3.40	1.16	−0.12	−0.06	−0.04	0.53^***^	−0.36^***^	−0.48^***^	1																
8 Day 2 workload	2.58	0.68	0.09	0.14	0.03	0.54^***^	−0.06	−0.14	0.36^***^	1															
9 Day 2 autonomous motivation	3.09	0.89	0.17^*^	0.19^*^	0.08	0.11	0.67^***^	0.60^***^	−0.20^*^	−0.02	1														
10 Day 2 workplace well-being	3.34	0.86	0.24^**^	0.18^*^	0.08	−0.02	0.50^***^	0.58^***^	−0.26^**^	−0.13	0.73^***^	1													
11 Day 2 emotional exhaustion	3.25	1.29	−0.15	−0.06	−0.08	0.36^***^	−0.29^***^	−0.41^***^	0.67^***^	0.54^***^	−0.20^*^	−0.29^***^	1												
12 Day 3 workload	2.60	0.63	0.02	0.04	−0.07	0.41^***^	−0.05	−0.19^*^	0.40^***^	0.57^***^	−0.00	−0.17^*^	0.49^***^	1											
13 Day 3 autonomous motivation	3.07	0.91	0.22^**^	0.23^**^	0.12	0.06	0.77^***^	0.70^***^	−0.28^***^	0.00	0.77^***^	0.67^***^	−0.21^*^	0.01	1										
14 Day 3 workplace well-being	3.17	0.86	0.24^**^	0.24^**^	0.09	−0.05	0.52^***^	0.59^***^	−0.30^***^	−0.18^*^	0.51^***^	0.68^***^	−0.33^***^	−0.12	0.71^***^	1									
15 Day 3 emotional exhaustion	3.31	1.29	−0.22^**^	−0.18^*^	−0.02	0.32^***^	−0.25^**^	−0.37^***^	0.58^***^	0.42^***^	−0.16	−0.33^***^	0.69^***^	0.53^***^	−0.26^**^	−0.38^***^	1								
16 Day 4 workload	2.59	0.66	−0.02	−0.02	0.12	0.19^*^	0.18^*^	0.02	0.27^***^	0.41^***^	0.09	−0.02	0.37^***^	0.58^***^	0.10	−0.05	0.44^***^	1							
17 Day 4 autonomous motivation	3.02	0.90	0.19^*^	0.22^**^	0.07	0.10	0.66^***^	0.59^***^	−0.25^**^	0.09	0.74^***^	0.56^***^	−0.14	0.17^*^	0.76^***^	0.55^***^	−0.13	0.21^*^	1						
18 Day 4 workplace well-being	3.22	0.81	0.17*	0.16*	0.09	0.02	0.54^***^	0.64^***^	−0.26^**^	−0.04	0.59^***^	0.66^***^	−0.23^**^	−0.01	0.63^***^	0.68^***^	−0.19^*^	0.01	0.71^***^	1					
19 Day 4 emotional exhaustion	3.21	1.36	−0.16*	−0.14	0.02	0.26^**^	−0.20^*^	−0.31^***^	0.56^***^	0.41^***^	−0.12	−0.22^**^	0.66^***^	0.48^***^	−0.14	−0.26^**^	0.76^***^	0.62^***^	−0.13	−0.26^**^	1				
20 Day 5 workload	2.57	0.71	0.04	−0.02	−0.05	0.30^***^	−0.11	−0.19*	0.33^***^	0.41^***^	−0.04	−0.16^*^	0.43^***^	0.48^***^	−0.06	−0.17^*^	0.41^***^	0.48^***^	0.05	−0.03	0.51^***^	1			
21 Day 5 autonomous motivation	2.98	0.90	0.22^**^	0.23^**^	0.10	0.04	0.74^***^	0.66^***^	−0.31^***^	0.01	0.77^***^	0.64^***^	−0.21^**^	0.04	0.83^***^	0.62^***^	−0.18^*^	0.10	0.83^***^	0.69^***^	−0.12	−0.05	1		
22 Day 5 workplace well-being	3.19	0.82	0.14	0.16^*^	0.14	−0.05	0.57^***^	0.58***	−0.23^**^	−0.07	0.53^***^	0.60^***^	−0.23^**^	0.03	0.60^***^	0.64^***^	−0.16^*^	0.09	0.56^***^	0.76^***^	−0.11	−0.13	0.71^***^	1	
23 Day 5 emotional exhaustion	3.24	1.44	−0.16^*^	−0.10	−0.06	0.32^***^	−0.22^**^	−0.29^***^	0.59^***^	0.47^***^	−0.13	−0.24^**^	0.77^***^	0.45^***^	−0.14	−0.24^**^	0.75^***^	0.47^***^	−0.09	−0.16*	0.77^***^	0.58^***^	−0.19^*^	−0.21^**^	1

N = 152. ^*^p < 0.05, ^**^p < 0.01, ^***^p < 0.001.

###### Hypothesis 1: the inverted U-shaped relationship between workload and workplace well-being

**Figure 3:**
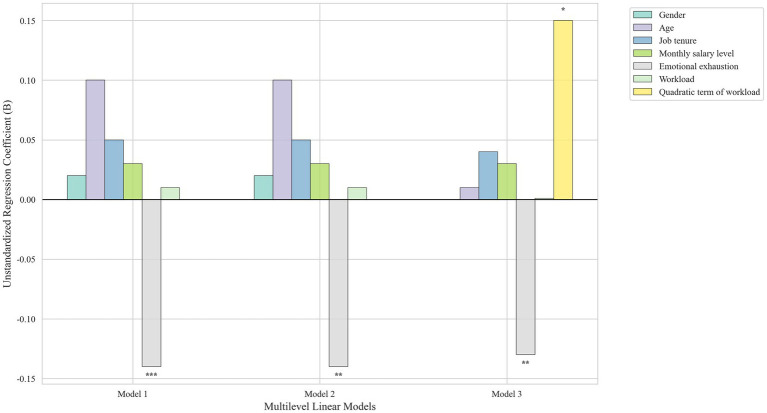
Results of multilevel linear models testing the inverted U hypotheses in Study 2.

**Figure 4:**
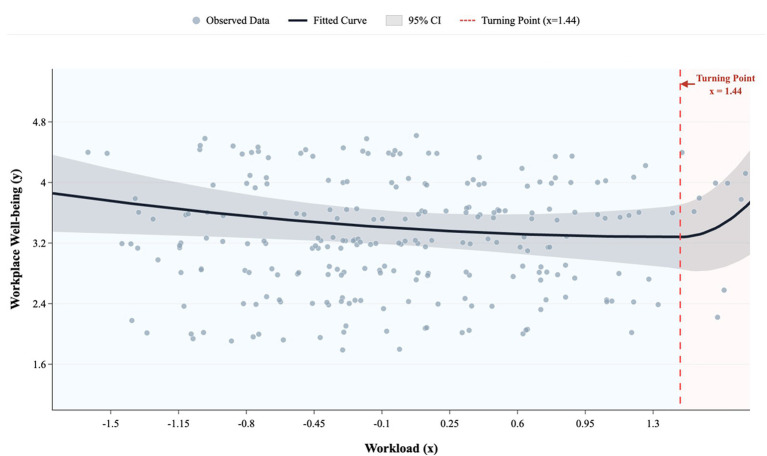
Positive U-curve relationship between workload and workplace well-being found in Study 2.

As shown in Table 4 and Figure 3, the linear and quadratic terms for workload in Study 2 were consistent with those in Study 1. Specifically, when both the linear and squared terms of workload were included in the model, the linear term for workload was not significant (*b = 0*.01, *p >* 0.05), but the quadratic term was the quadratic term was positive and approached marginal significant (*b =* 0.15, *p =* 0.07), indicating a curvilinear relationship between workload and workplace well-being. The result was the opposite of what was predicted by Hypothesis 1. Rather than there being an inversed U-shaped curve, there was a positive U-curve relationship, such that workplace well-being was lower at moderate levels of workload and higher at both low and high levels of workload (see Figure 4).

**Table 4:** Results of multilevel linear models testing the inverted U hypotheses in Study 2.

Variable	Model 1			Model 2			Model 3		
	*b*	SE	95%CI	*b*	SE	95%CI	*b*	SE	95%CI
Gender	0.02	0.11	(−0.16, 0.19)	0.02	0.11	(−0.16, 0.19)	0.00	0.11	(−0.16, 0.19)
Age	0.01	0.00	(−0.00, 0.02)	0.01	0.01	(−0.00, 0.02)	0.01	0.01	(−0.00, 0.02)
Job tenure	0.05	0.05	(−0.08, 0.18)	0.05	0.08	(−0.08, 0.18)	0.04	0.08	(−0.08, 0.18)
Monthly salary level	0.03	0.03	(−0.01, 0.07)	0.03	0.02	(−0.01, 0.07)	0.03	0.02	(−0.01, 0.07)
Emotional exhaustion	−0.14^***^	0.04	(−0.20, −0.08)	−0.14^**^	0.04	(−0.21, −0.08)	−0.13^**^	0.04	(−0.21, −0.08)
Workload				0.01	0.07	(−0.10, 0.13)	0.00	0.06	(−0.09, 0.12)
Quadratic term of workload							0.15^+^	0.04	(−0.00, 0.31)

*N* = 152. Unstandardized regression coefficients are shown. + *p* < 0.1, ** *p* < 0.01, *** *p* < 0.001.

###### Hypothesis 2: The mediation effect of autonomous motivation

Results indicated that the direct effect of workload on workplace well-being was not statistically significant (*b* = 0.04, 95% CI = [−0.05, 0.13]). In addition, the bootstrapped indirect effect of workload on workplace well-being through autonomous motivation was also not significant (*b* = 0.13, 95% CI = [−0.21, 0.48]). Taken together, the results did not support Hypothesis 2.

#### Supplementary analyses 1: spline and piecewise checks

##### Restricted cubic spline analyses

Study 2 further examined the non-linear relationship between workload and workplace well-being using restricted cubic spline regression implemented in R 4.0.0. A linear mixed-effects model (y ~ Mx + control variables) was first estimated as the baseline specification. This model was then compared with a mixed-effects model incorporating a natural spline term for workload with two-degrees-of-freedom (y ~ ns (Mx, *df* = 2) + control variables). Model comparison based on the AIC indicated that the spline model provided a slightly better fit than the linear model (AIC = 1478.83). Although the individual spline basis terms were only marginally significant (*p* = 0.06 and *p* = 0.08, respectively), the overall improvement in model fit provides suggestive evidence of a non-linear association between workload and workplace well-being.

##### Piecewise mediation analysis

To further explore the functional form of this non-linear relationship and its potential underlying mechanism, a piecewise mediation approach was conducted. The analytical procedure consisted of three steps: (a) identifying a potential turning point in the relationship between workload and workplace well-being, (b) segmenting the sample based on this turning point, and (c) estimating the mediation paths separately for each segment.

The turning point was identified using locally weighted regression (LOESS) combined with model fit comparison based on AIC. This procedure suggested a turning point at Mx = 1.44. The piecewise model showed improved fit relative to the linear model (AIC = 1478.54 vs. 1484.72). Consistent with this pattern, the quadratic term for workload was statistically significant (*β* = 0.13, *p* = 0.03), further supporting the presence of non-linearity.

Piecewise regression results indicated that when workload was below the estimated turning point (Mx < 1.44), workload was negatively associated with workplace well-being (*β* = −0.09, *SE* = 0.04, *p* = 0.03). Above the turning point (Mx ≥ 1.44), the slope became positive (*β* = 4.30, *SE* = 1.44, *p* = 0.003). This pattern is consistent with a shift from a negative to a positive association, a defining characteristic of a positive U-shaped relationship.

However, it should be noted that the number of observations above the turning point was very small (*n* = 2), which substantially limits the precision and generalizability of slope estimates in the high-workload range. Accordingly, the piecewise results should be interpreted as exploratory and complementary to the spline-based analyses.

In the linear mixed-effects mediation model controlling for covariates, workload did not significantly predict autonomous motivation (*β* = −0.02, *SE* = 0.03, *p* = 0.62), whereas autonomous motivation was positively related to workplace well-being (*β* = 0.49, *SE* = 0.04, *p* < 0.001). The indirect effect of workload on workplace well-being via autonomous motivation was not significant. This pattern is consistent with the multilevel mediation results reported in Study 2 and suggests that autonomous motivation does not mediate the overall linear association between workload and workplace well-being.

#### Supplementary analyses 2: robustness

We performed a similar robustness check in Study 2 by excluding emotional exhaustion. The results showed that the quadratic effect of workload on workplace well-being remained positively significant (*β =* 0.19, *p =* 0.016). In the mediation test with autonomous motivation as the outcome, the quadratic term of workload was marginally significant (*β =* 0.06, *p* = 0.096). The indirect effect via autonomous motivation was not statistically significant (effect = 0.16, 95% CI [−0.05, 0.36]). These findings align with the results of the primary models, supporting the reliability of the positive U-shaped curve between workload and workplace well-being observed in Study 2.

## Discussion

The relationship between workload and workplace well-being has been unclear. We tested a non-linear relationship between workload and workplace well-being, as well as the mediating role of autonomous motivation. We originally hypothesized an inverted U-shaped relationship, such that workplace well-being would peak at moderate levels of workload. Contrary to this expectation, results from both studies provided evidence for a non-linear pattern characterized by positive curvature. Specifically, the supplementary analyses indicated that increases in workload from low to moderate levels were associated with lower workplace well-being, whereas increases from moderate to higher levels were associated with higher workplace well-being.

A potential explanation for this U-curve relationship is that workload induces workplace well-being via a process of that creates an internal equilibrium. When the workload level increases from low to moderate, employees’ internal equilibrium might not be evoked. On one hand, employees who experience some level but non-zero workload have to handle that workload. However, that relative lower level workload which contains less job requirements and demands might have less opportunity for personal development and achievement. One the other hand, increasing workload from zero to low to moderate level would decline employees’ discretionary time. The Less discretionary time may have a diminishing effect on well-being (e.g., Sharif et al., 2021). Hence, Employees would have to cope with a low but non-zero workload, which might deplete resources and promote less competence and autonomy.

An increase in workload from moderate to high might evoke an equilibrium process. Workload at the high end of the spectrum may lead to a disturbance in employees’ internal equilibrium. At this point, the workload becomes a challenge, prompting the individual to creatively cope with the challenge. In the process of reintegration, individuals may experience development, such as greater self-esteem and self-efficacy. This growth, in turn, may increase autonomous motivation and workplace well-being (Richardson et al., 1990).

There was mixed evidence of autonomous motivation being a mediator of the association between workload and workplace well-being. In Study 1, the mediating effect was significant, while in Study 2, no significant mediation was detected. One possible explanation for the inconsistent results between Study 1 and Study 2 is the differences in age and tenure. The participants in Study 1 were mainly 36 years or above, and their average job tenure was generally more than 5 years. In Study 2, the participants were mainly under 25 years old, and their average job tenure was less than 3 years. The age difference may lead to a difference in work motivation. Twenge (2010) found that the younger generation’s intrinsic work values showed a downward trend, while extrinsic work values showed an upward trend. To some extent, younger employees’ autonomous motivation may be weaker than their extrinsic motivation. In addition, an increase in length of service may also increase employee loyalty (Ye et al., 2016), and stimulate their endogenous motivation. Researchers could explore that generational difference in the future.

### Implications

Almost all existing studies have assumed and only tested linear relations between workload and workplace well-being, and the results were inconsistent. Our study extends previous research by considering the non-linear relation between workload and workplace well-being. The non-linear relations between workload and workplace well-being would be especially beneficial to explain the mixed results about workload → workplace well-being. Our work indicates the importance of considering the effects of increasing workload on workplace well-being, especially for those who are relatively low in workload. This extends STD literature by considering the changes in workplace context that may impact employees’ autonomous motivation and workplace well-being. Although employees’ workplace well-being can be improved when their autonomous motivations are invoked in workplace (Gagné and Deci, 2005), their personal working states (i.e., workload level) should also be considered.

The current research also adds evidence about the non-linear effects in well-being research. There is a debate about the U-shaped effects in well-being research (Ren et al., 2022). Specifically, while some studies did not find a non-linear association between various predicators and psychological well-being, including conscientiousness (Nickel et al., 2019), self-control (Wiese et al., 2018), the recent research did find a non-linear effect in psychological well-being (Ren et al., 2022). Our work adds evidence about the U-curve effect in well-being research. Furthermore, we extend the literature by studying a workplace predictor of well-being: the workload. In addition, the present research adds to knowledge in workload research area. A previous research found an inverted U-shape relationship between workload and task performance (Bruggen, 2015). Here, we found a U-shape relationship between workload and workplace well-being. That is, the U-shaped effects of workload is likely distinct when considering different outcomes.

Finally, our findings may have practical value for organizations and managers who are trying to promote employees’ workplace well-being. Managers should identify employees whose existing levels of workload are moderate, and identify strategies that may improve their autonomous motivation and workplace well-being. These strategies would not need to be implemented with the employees whose workloads are high. When dealing with a high level workload, employees experience increasing autonomous motivation and workplace well-being.

### Limitations and future directions

Despite its contributions, the present research has a few limitations. Firstly, although our experience-sampling design (Study 2) to some extent can help to establish causality, our analyses in both studies were based on questionnaire data. A laboratory experiment that manipulates workload would strengthen the ability to make causal inferences. For example, Sherf et al. (2019) manipulated workload by asking participants to complete a task under different time limits. In addition, because our sample was predominantly made up of young adults, future research might examine the generalizability of the findings to an older population or document key differences between older and younger employees.

Second, other mechanisms of the association between workload and workplace well-being should be investigated in the future. Although we used SDT perspective to hypothesize the non-linear relations between workload and workplace well-being, we did not explicitly test some of the mechanisms reasoned from the theory. In particular, we did not measure employees’ satisfaction of fundamental psychological needs, although previous research has suggested that satisfaction of fundamental psychological needs, such as autonomy, impact employees’ well-being (e.g., Gagné and Deci, 2005; Kuykendall et al., 2020). Future research may benefit from testing the non-linear relations between workload, satisfaction of fundamental psychological needs and workplace well-being.

## Conclusion

What is the relationship between workload and workplace well-being? To answer this question, we tested the possibility that the relationship is non-linear. The results of a questionnaire study and an experience sampling study indicated a positive U-curve relationship: increasing workload from low to moderate decreases workplace well-being, but increasing workload from moderate to high improves it.

## Data Availability

All research materials, data, and analysis scripts are available on the Open Science framework (OSF): https://osf.io/672xc/?view_only=15b146b2074240afbf2de48d4408bf4c.
